# Sodium 2-Mercaptoethanesulfonate (MESNA), Ifosfamide, Mitoxantrone, and Etoposide (MINE) in Transplant-Ineligible Relapsed/Refractory Diffuse Large B-Cell Lymphoma: Is the Old Regimen Still Gold?

**DOI:** 10.7759/cureus.87128

**Published:** 2025-07-01

**Authors:** Leonardo Maia Moço, Ana Maria Hortas, Inês Ramos, Alice Fontoura, Gonçalo de Câmara Negalha, Gil Brás, Mário Mariz

**Affiliations:** 1 Department of Hematology and Bone Marrow Transplantation, Instituto Português de Oncologia do Porto Francisco Gentil, Porto, PRT; 2 Laboratory of Histology and Embryology, Department of Microscopy, School of Medicine and Biomedical Sciences, Universidade do Porto (ICBAS-UP), Porto, PRT; 3 Hematopoiesis and Microenvironments Group, Instituto de Investigação e Inovação em Saúde, Universidade do Porto (i3S-UP), Porto, PRT; 4 Department of Clinical Hematology, Centro Hospitalar de Trás-Os-Montes e Alto Douro, Vila Real, PRT

**Keywords:** autologous hematopoietic stem cell transplantation, diffuse large b-cell lymphoma (dlbcl), etoposide, ifosfamide, mitoxantrone, non-hodgkin lymphoma, systemic chemotherapy

## Abstract

Introduction: For decades, the rituximab, cyclophosphamide, vincristine, doxorubicin, and prednisolone (R-CHOP) regimen has been the standard treatment for aggressive B-cell non-Hodgkin lymphoma (NHL), such as diffuse large B-cell lymphoma (DLBCL). However, patients with relapsed or refractory (R/R) disease continue to face a poor prognosis. Those eligible for autologous hematopoietic stem cell transplantation (ASCT) are usually rescued with a platinum-containing regimen. Conversely, milder regimens are preferred for ineligible patients, such as gemcitabine and oxaliplatin (GemOx). At our institution, the standard second-line treatment for patients over 65 years or with comorbidities that make them unsuitable for ASCT is a non-platinum-based regimen composed of sodium 2-mercaptoethanesulfonate (MESNA), ifosfamide, mitoxantrone, and etoposide (MINE). Although newer targeted and immune-based therapies are emerging, there remains a lack of prospective studies on optimal treatment choices for this group of patients, particularly regarding non-platinum-based regimens.

Aim: The study aimed to evaluate in a real-world setting the efficacy and safety profile of MINE, with or without rituximab, in patients with R/R DLBCL.

Methods: This was a retrospective, single-center study conducted from April 2007 to August 2024. It included patients who underwent at least one cycle of MINE. Data were collected from patients' electronic records. The primary endpoints were overall survival (OS), progression-free survival (PFS), and duration of response (DoR). The secondary endpoints included complete response (CR) and overall response rates (ORR), as well as toxicity-related surrogates, such as the total number of required RBC units or platelet concentrates (PC), total number of febrile neutropenia (FN) episodes, ICU admissions, and treatment-emergent adverse events (TEAEs).

Results: A total of 167 patients were included, with a median follow-up time of 10 months (IQR: 4-40), a female-to-male ratio of 1.26, and 127/167 (76.0%) patients over the age of 65 (median age: 70). About 47/167 (28.1%) cases resulted from the transformation of indolent NHL. Disease was localized in 94/151 (62.3%), and 52/166 (31.3%) were refractory to the previous treatment. The protocol was used as second-line therapy in 121/167 (72.4%), with rituximab added in 86/167 (51.5%). Median OS, PFS, and DoR were 11, seven, and 24 months, respectively. In univariate analysis, PFS and OS were significantly higher in patients receiving rituximab with MINE and lower in those who presented with bulky masses, advanced-stage disease, and refractoriness to the prior line. CR was 45.3% and ORR was 63.5%. Myelotoxicity was the primary complication, with 38/124 (30.6%) patients developing FN, and six (4.8%) requiring ICU admission. This was followed by cardiotoxicity in 18/124 (14.5%). Treatment was discontinued in 17/152 (11.2%) patients, and 11/152 (7.2%) died due to toxicity.

Conclusions: This retrospective study demonstrates that the MINE protocol offers favorable outcomes and an acceptable safety profile, with myelotoxicity as the most significant adverse effect. Although inclusion criteria were not strictly limited to ineligible patients, they constituted the majority of this cohort. In conclusion, while additional prospective studies are needed, these findings reinforce MINE as a still viable and cost-efficient alternative, particularly for R/R DLBCL patients who are not eligible for ASCT.

## Introduction

Diffuse large B-cell lymphoma (DLBCL) is the most common subtype of aggressive non-Hodgkin lymphoma (NHL), accounting for approximately one-third of all NHL cases [1]. The first-line treatment typically involves chemoimmunotherapy with rituximab plus cyclophosphamide, vincristine, doxorubicin, and prednisolone (R-CHOP). Like most aggressive NHLs, DLBCL demonstrates significant remission rates, with five-year survival rates ranging from 60% to 70%. However, approximately 20-40% of patients are refractory or experience relapse. This group of patients has traditionally been associated with a dismal prognosis. Fortunately, the treatment landscape for DLBCL is evolving, with recent advances leading to improved outcomes [2].

For patients eligible for autologous stem cell transplantation (ASCT), salvage regimens usually include a platinum-based therapy. However, the majority of DLBCL patients are older and often have comorbidities that render them unfit for intensive chemotherapy. In these cases, less intensive regimens are preferred, such as gemcitabine and oxaliplatin (GemOx), or even non-platinum-based regimens such as sodium 2-mercaptoethanesulfonate (MESNA), ifosfamide, mitoxantrone, and etoposide (MINE), with or without rituximab [3]. At our institution, rituximab plus MINE (R-MINE) is the preferred option for patients who are older than 65 years or considered ineligible for ASCT due to incompatible comorbidities. However, it may also be considered in subsequent lines, on an individualized basis.

Regarding non-platinum-based regimens, they are less commonly used considering the cumulative cardiotoxicity from anthracyclines and mitoxantrone, the significant myelosuppression associated with high-dose ifosfamide, as well as the emergence of newer therapies. In spite of that, evidence regarding these regimens is very limited and perhaps worth consideration. A retrospective study involving 92 patients with relapsed or refractory (R/R) NHL treated with a maximum of six cycles of MINE, followed by consolidation with etoposide, methylprednisolone, high-dose cytarabine, and cisplatin (ESHAP), reported complete response (CR) and overall response rate (ORR) of 48% and 69%, respectively. The median overall survival (OS) was 24 months, and the median time to treatment failure was 12 months [4]. Another retrospective study, conducted on 44 patients under 60 years with R/R aggressive NHL who were treated with an intensive salvage regimen consisting of high doses of ifosfamide, etoposide and mitoxantrone, followed by ASCT, demonstrated a CR of 41%, with two-year progression-free survival (PFS) and OS rates of 38% and 52%, respectively, after a median follow-up of 52 months. Myelosuppression was typically the most serious complication, usually profound but transient, with a median duration of absolute neutrophil count less than 0.5 x 10^9^/L of six days (range 3-12 days) [5]. Finally, a smaller study involving 27 patients with R/R DLBCL treated with three cycles of rituximab plus a combination of ifosfamide, epirubicin, and etoposide (IVE), or MINE for patients older than 65, reported a median OS of 32 months after a median follow-up of 28 months [6].

Therefore, additional randomized, prospective, and large-scale studies are warranted to validate these findings and confirm whether non-platinum-based regimens remain a viable treatment option in the current therapeutic landscape. To help fill this gap, this study aimed to evaluate the efficacy and safety profile of the MINE protocol (with or without rituximab) in patients with R/R DLBCL ineligible for ASCT.

## Materials and methods

Patients

This retrospective, single-center study included patients treated for R/R DLBCL with at least one cycle of the MINE protocol (with or without rituximab) between April 2007 and August 2024. The treatment scheme is depicted in Table 1 [4]. Cases of NHL transformed from indolent subtypes were also considered. Demographic, disease-related, and treatment-related data were collected from patients’ electronic medical records. Tumor masses measuring 10 cm were classified as bulky. Refractoriness to the previous line of treatment also included patients who relapsed within the first six months after completing therapy. Risk scores were solely calculated at the time of diagnosis; therefore, the Revised International Prognostic Index (R-IPI) and the age-adjusted International Prognostic Index (aaIPI) were not applied to cases of transformed NHL. This study was approved by the institutional ethics board of Instituto Português de Oncologia do Porto Francisco Gentil on January 22, 2025 (reference CES.020_25).

**Table 1:** Schematic overview of the R-MINE protocol. Standard treatment typically consists of six 21-day cycles. IV: intravenous; MESNA: sodium 2-mercaptoethanesulfonate; X: drug administered on the given day; R-MINE: rituximab plus MESNA, ifosfamide, mitoxantrone, and etoposide; D1: day 1; D2: day 2; D3: day 3 Adapted from Rodriguez et al. [4]. No copyrighted material from the original article has been reused.

Drug	D1	D2	D3
Rituximab (375 mg/m^2^ IV)	X		
Ifosfamide (1330 mg/m^2^ IV)	X	X	X
MESNA (1330 mg/m^2^ IV together with ifosfamide >400 mg IV, four hours after ifosfamide)	X	X	X
Mitoxantrone (8 mg/m^2^ IV)	X		
Etoposide (65 mg/m^2^ IV)	X	X	X

Endpoints

The primary endpoints included OS, defined as the time from the start of treatment to death from any cause; PFS, defined as the time from the start of treatment to disease progression or death from any cause; and, finally, duration of response (DoR), defined as the time from the start of treatment to disease progression or death from any cause among patients who achieved at least a partial response (PR) to the MINE protocol. Secondary endpoints included CR, ORR, and toxicity-related surrogates. These toxicity metrics included the total number of RBC units or platelet concentrates (PC) transfused, total number of febrile neutropenia (FN) episodes, ICU admissions, and treatment-emergent adverse events (TEAEs).

Statistical analysis

For descriptive analysis, qualitative variables were expressed as proportions, and where relevant, as maximum and minimum values. Continuous variables were reported as medians and IQR. Subgroup analysis categorized age into <70 and ≥70 years and extranodal involvement into <2 and ≥2 sites. Observational periods were censored at the date of the last contact if no event was observed. Kaplan-Meier curves were constructed to estimate survival, and multivariate Cox regression analysis was performed using time to death or progression as the dependent variable. All statistical analyses were conducted using IBM SPSS Statistics v29.0.0.0 software (IBM Corp., Armonk, USA).

## Results

Descriptive analysis

Between April 2007 and August 2024, 167 patients with R/R DLBCL were treated with the MINE protocol (Table 2). The female-to-male ratio was 1.26, and the median age was 70 years (IQR 66-74), while only 40/167 (24.0%) patients were 65 years or less. The most common histological subtype was DLBCL not otherwise specified (NOS) in 111/167 (66.5%) patients. About 47/167 (28.1%) patients had DLBCL transformed from a prior indolent NHL. Although 94/151 (62.3%) patients presented with localized disease, lactate dehydrogenase (LDH) levels were elevated in 96/150 (64.0%) patients, and 66/109 (60.6%) had a poor-risk R-IPI score. Additionally, 52/166 (31.3%) patients were refractory to their previous line of treatment.

**Table 2:** Descriptive analysis of baseline demographic and disease-related characteristics. Some clinical and laboratory parameters at diagnosis were missing because electronic medical records have only been available at our institution since 2012. DLBCL: diffuse large B-cell lymphoma; EBV: Epstein-Barr virus; GCB: germinal center B-cell; LDH: lactate dehydrogenase; R-IPI: Revised International Prognostic Index; NOS: not otherwise specified; B symptoms: fever, night sweats, and weight loss; aaIPI: age-adjusted International Prognostic Index

Characteristic	n	(%)
Female-to-male ratio	1.26	
Age - median (IQR)	70	(66-74)
Histological subtype		
DLBCL	167	
NOS	111	-66.5
Immune-privileged sites	4	-2.4
EBV+	1	-0.6
T-cell/histiocyte-rich	4	-2.4
Transformation	47	-28.1
Bulky mass (12 missing)	42	-27.1
B symptoms (24 missing)	58	-40.6
Stage (16 missing)		
Localized	94	-62.3
Advanced	57	-37.7
Extranodal involvement 2 or more (20 missing)	36	-24.4
Increased LDH (17 missing)	96	-64
R-IPI (58 missing)		
Very good	4	-3.6
Good	39	-35.8
Poor	66	-60.6
aaIPI (58 missing)		
Low	12	-11
Low-intermediate	21	-19.3
High-intermediate	68	-62.4
High	8	-7.3
Hans algorithm (88 missing)		
GCB	41	-51.9
Non-GCB	38	-48.1
Response to previous line (1 missing)		
Refractory	52	-31.3
Sensitive	114	-68.7
Prior rituximab	155	-92.8

Most patients (155/167, 92.8%) had prior exposure to rituximab, and the ratio of MINE with rituximab to MINE without rituximab was nearly 1:1. About 81/167 (48.5%) patients were not treated with rituximab for two main reasons: (i) rituximab was not included in the department protocol at the time for 41/81 (50.6%) patients, and (ii) the remaining 40/81 (49.4%) patients had received rituximab within the preceding six months. Almost three-quarters of patients (121/167, 72.4%) received this protocol as second-line treatment, and half of them (80/162, 49.4%) completed the full six cycles. Among the patients who received MINE as second-line treatment, 14/121 (13.2%) were younger than 65. 

Only a minority underwent further consolidation treatment, mainly with radiotherapy. Two patients out of 167 (1.2%) who were treated with MINE in a third-line setting were still eligible for consolidation with ASCT. Recently, our institution has also started using R-MINE as a bridging therapy for patients with NHL who are candidates for chimeric antigen receptor (CAR) T-cell therapy (2/167, 1.2%). Treatment-related characteristics are summarized in Table 3.

**Table 3:** Descriptive analysis of treatment-related characteristics. ASCT: autologous stem cell transplantation; CAR T: chimeric antigen receptor T-cell; ESHAP: etoposide, methylprednisolone, high-dose cytarabine, and cisplatin; MINE: sodium 2-mercaptoethanesulfonate (MESNA), ifosfamide, mitoxatrone, etoposide; RT: radiotherapy

Characteristic	n	(%)
MINE protocol		
Without rituximab	81	-48.5
With rituximab	86	-51.5
As a bridge to CAR T	2	-1.2
Line of MINE		
2^nd^	121	-72.4
3^rd^	35	-21
4^th^ or more	11	-6.6
No. of cycles (5 missing)		
1	22	-13.6
2	14	-8.6
3	24	-14.8
4	15	-9.3
5	7	-4.3
6	80	-49.4
Consolidation		
ESHAP	4	-2.4
No. of cycles (IQR)	4	(1-6)
ASCT	2	-1.2
RT	21	-12.6

Outcomes

**Figure 1:**
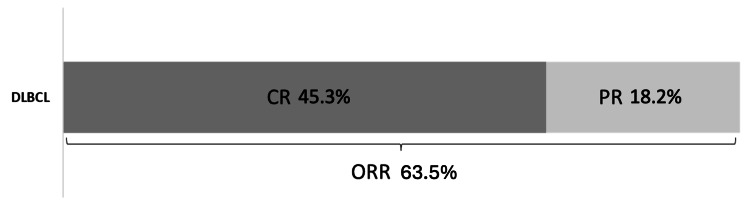
Bar chart displaying the proportions of CR and PR. CR: complete response; PR: partial response; ORR: overall response rate

Over a median follow-up period of 10 months (IQR 4-40), the CR rate was 45.3% (67/148) and ORR was 63.5% (94/148) (Figure 1). However, 115/156 (73.7%) patients experienced R/R disease following MINE treatment. Patients who did not undergo a response assessment or who died too early during the treatment course to determine refractoriness were not considered for these proportions, respectively. Regarding survival, 134/167 (80.2%) patients ultimately died. The cause of death could not be clearly determined in six patients who died at other institutions. Among the remaining 128 patients, the majority of deaths (103/128, 80.5%) were attributed to disease progression. 

**Figure 2:**
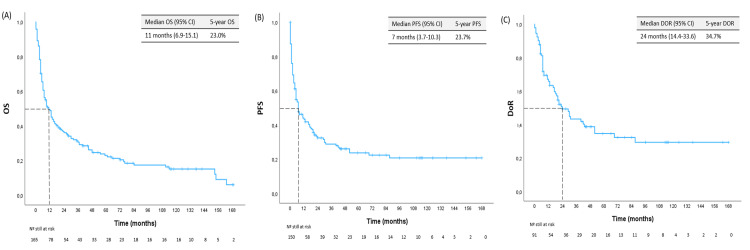
Survival curves regarding (A) OS, (B) PFS, and (C) DoR. DoR: duration of response; PFS: progression-free survival; OS: overall survival

Survival analysis (Figure 2) revealed a median OS of 11 months (95% CI: 6.9-15.1), a median PFS of seven months (95% CI: 3.7-10.3), and finally a median DoR of 24 months (95% CI: 14.4-33.6). 

The potential impact of baseline characteristics on the primary endpoints was also assessed. In univariate analysis, several factors were identified as risk factors for worse outcomes. Advanced-stage disease and not receiving rituximab were associated with worse OS, PFS, and DoR, while bulky disease and refractoriness to prior treatment were only associated with worse OS and PFS, as depicted in Table 4. However, all these eventual risk factors lost statistical significance in multivariate analysis. Other variables, including age, presence of B symptoms (fever, night sweats, and weight loss), LDH levels, extranodal involvement, R-IPI score, and Hans algorithm, did not show a significant impact on outcomes. 

**Table 4:** OS, PFS, and DoR (in months) regarding the presence or absence of clinical variables identified as possible risk factors in univariate analysis. DoR: duration of response; PFS: progression-free survival; OS: overall survival; NA: not applicable

Risk factors	OS (months)	PFS (months)	DoR (months)
Bulky disease	3 (95% CI: 1.5-4.5) *vs* 18 (95% CI: 4.5-31.5)	0 *vs* 7 (95% CI: 0.0-14.7)	NA
$\rho$-value	0.004	0.021	NA
Advanced stage	4 (95% CI: 2.7-5.4) *vs* 22 (95% CI: 5.0-39.0)	0 *vs* 12 (95% CI: 0.82-23.2)	4 (95% CI: 2.5-5.5) *vs* 37 (95% CI: 12.7-61.3)
$\rho$-value	<0.001	<0.001	0.001
Refractoriness to prior treatment	3 (95% CI: 1.7-4.3) *vs* 19 (95% CI: 7.7-30.3)	0 *vs* 11 (95% CI: 4.8-17.2)	NA
$\rho$-value	0.014	0.003	NA
Rituximab	26 (95% CI: 7.4-44.6) *vs* 4 (95% CI: 2.5-5.5)	21 (95% CI: 9.4-32.6) *vs* 0	37 (95% CI: 12.4-61.6) *vs* 3 (95% CI: 1.2-4.8)
$\rho$-value	<0.001	<0.001	0.003

Safety profile

Myelotoxicity emerged as the most prominent complication associated with MINE. As expected, cardiotoxicity was also observed, albeit to a much lesser extent. The transfusional burden was substantial, with 57/124 (46%) patients requiring at least one RBC unit and 17/124 (13.7%) needing at least one PC. Approximately one-third of patients (38/124, 30.6%) experienced FN, and six out of 124 (4.8%) cases required transfer to the ICU. Moreover, 17/152 (11.2%) patients discontinued treatment due to toxicity, and 11/152 (7.2%) succumbed to complications, primarily from myelosuppression and infection. TEAEs are described in detail in Table 5. Additionally, at least two out of 167 (1.2%) patients developed possible secondary myelodysplastic syndrome following MINE treatment, though causality could not be definitively established.

**Table 5:** Transfusional burden and TEAEs registered during treatment with R-MINE. FN: febrile neutropenia; PC: platelet concentrate; TEAEs: treatment-emergent adverse events; R-MINE: rituximab plus sodium 2-mercaptoethanesulfonate (MESNA), ifosfamide, mitoxantrone, and etoposide

Toxicity	n	(%)
Myelotoxicity (43 missing)		
No. of patients who needed RBC unit(s)	57	-46
No. of patients who needed PC	17	-13.7
No. of patients who had FN	38	-30.6
No. of patients who were admitted to ICU	6	-4.8
Other toxicities		
Cardiotoxicity	6	-4.8
Nephrotoxicity	2	-1.6
Hepatotoxicity	1	-0.8
TEAEs leading to non-elective or prolonged admission(s) (44 missing)	45	-36.4
TEAEs leading to dosage adjustment or cycle(s) postponed (47 missing)	37	-30.8
TEAEs leading to discontinuation (15 missing)	17	-11.2
TEAEs leading to death (15 missing)	11	-7.2
Causes of death		
Infection	9	-81.8
Cardiotoxicity	1	-9.1
Not clarified	1	-9.1

## Discussion

According to the institutional protocol, MINE is only used as a second-line therapy in patients over 65 years of age and/or those deemed ineligible for ASCT. In our series, 127/167 (76.0%) patients were older than 65. Among the 121 patients who received MINE as second-line therapy, 107/167 (64.1%) were also over 65. For the remaining 14 patients, it is reasonable to assume that they were deemed unsuitable for ASCT due to comorbidities. Only 2/167 (1.2%) patients underwent ASCT after MINE, which constituted a third-line salvage option after failure of a second-line platinum-containing regimen. In summary, although comorbidities-related data were not fully ascertained, 76.0% of the patients were unequivocally ineligible for ASCT in light of our institutional protocol at least due to age, and 64.1% in a second-line treatment setting. Therefore, patients at first relapse who were over 65 and/or ASCT-ineligible clearly represent the primary setting of this study. Nonetheless, treatment eligibility is increasingly guided by biological rather than chronological age. The decision to exclude potentially fit patients over 65 from ASCT solely on the basis of age may, therefore, indirectly overestimate the observed outcomes in our cohort.

In a vast majority of centers, the gold-standard second-line treatment for R/R DLBCL traditionally comprises salvage chemotherapy with platinum-based regimens, followed by ASCT for eligible patients. For non-eligible patients, apart from R-MINE, rituximab plus GemOx (R-GemOx) remains a common salvage regimen, despite its limitations. A recent real-life study involving 196 ineligible patients reported, at the end of treatment with R-GemOx, a CR and ORR of 33% and 38%, respectively, with median OS and PFS of 10 and five months, respectively [7]. These findings are further supported by the more recently published multicenter retrospective Lymphoma Epidemiology of Outcomes (LEO) cohort study (NCT02736357) [8]. However, this regimen is also associated with significant utilization of healthcare services and, therefore, higher costs [9]. 

Nonetheless, recent advancements have introduced promising alternatives. Namely, CAR T-cell therapy, such as axicabtagene ciloleucel (axi-cel) (ZUMA-7 trial) and lisocabtagene maraleucel (TRANSFORM trial), is now approved by both the FDA and EMA as the recommended second-line options for patients with primary refractory disease or early relapses (within 12 months) as well as in later lines of therapy [10,11]. Contrarily, for younger patients with a late first relapse, conventional platinum-based salvage chemoimmunotherapy followed by consolidative ASCT remains the only available option. Moreover, axi-cel was also studied as a second-line option in transplant-ineligible patients, in the phase II clinical trial ALYCANTE, obtaining a CR at three months post-CAR T-cell infusion of 71% and a median PFS of 11.8 months, at a median follow-up time of 12 months. The median OS was still not reached [12]. Tisagenlecleucel, in turn, is currently indicated only after two prior lines of therapy, under the JULIET trial [13]. However, results from the recent phase III BELINDA trial so far suggest that its efficacy in earlier lines remains suboptimal [14]. 

Beyond CAR T-cell therapy, bispecific antibodies targeting both CD3 and CD20, such as epcoritamab, odronextamab, and glofitamab, have demonstrated encouraging results and are both Food and Drug Administration (FDA) and European Medicines Agency (EMA)-approved for patients with R/R DLBCL after two prior lines of therapy [3,15-18]. Recently, some bispecific antibodies have been explored in association with GemOx, also with quite optimistic results [19,20]. While these immunotherapies hold promise, their associated adverse effects, including cytokine release syndrome, immune effector cell-associated neurotoxicity syndrome mainly after CAR T-cell therapy, prolonged and late-onset cytopenias, and recurrent infections, may limit their use, particularly in older/frail patients. 

Therefore, in cases of primary refractoriness or first relapse where neither ASCT nor CAR T-cell therapy are viable options, other emerging milder regimens should be examined in detail. Tafasitamab (a monoclonal antibody targeting CD19) plus lenalidomide demonstrated excellent results in the setting of the single-arm phase II L-MIND trial, which included a total of 80 patients. They achieved a median OS of 33.5 months (95% CI: 18.3-not reached (NR)), a median PFS of 11.6 months (95% CI: 5.7-45.7), a CR of 41.3% (95% CI: 30.4-52.8) and an ORR of 57.5% (95% CI: 45.9-68.5) [21]. Nonetheless, this trial excluded primary refractory patients and those who had undergone more than three lines of therapy [22]. In fact, real-world analyses reveal a significant discrepancy in outcomes [23-25]. Another approved regimen, rituximab plus bendamustine and polatuzumab (R-Pola-Benda) (an antibody-drug conjugate (ADC) targeting CD79b and delivering the anti-mitotic agent monomethyl auristatin E) achieved a median OS of 12.5 months (95% CI: 8.2-23.1), a median PFS of 6.6 months (95% CI: 5.1-9.2), and a median DoR of 9.5 months (95% CI: 7.9-12.1) in a phase Ib/II randomized clinical trial, including 152 patients in the experimental arm. The CR was 38.7% (95% CI: 29.4-48.6) and ORR 41.5% (95% CI: 45.8-77.3) [26]. Finally, the multicenter, open-label, phase II, single-arm clinical trial LOTIS-2 evaluated loncastuximab-teserine (an ADC targeting CD19 with an alkylating payload of pyrrolobenzodiazepine) in a pool of 145 patients who had already been submitted to at least two prior lines. Median OS was 9.5 months (95% CI: 6.7-11.5), median PFS was 4.9 months (95% CI: 2.9-8.3), CR was 24.8%, and ORR was 48.3%. Patients who obtained a CR showed durable responses, having not reached median OS and PFS yet [27]. Despite the absence of robust randomized, prospective studies directly comparing MINE with these regimens, and also with each other, our findings raise the possibility that MINE, especially when in association with rituximab, might be, at least, non-inferior to R-GemOx and R-Pola-Benda. 

As expected, myelotoxicity was the most common adverse effect in our cohort, followed by cardiotoxicity in six out of 124 patients (4.8%). Despite the acceptable cumulative dose of mitoxantrone, these patients were typically older and often presented with cardiovascular comorbidities. Additionally, prior exposure to doxorubicin was nearly universal in this population. Although somewhat controversial, most of the literature assumes that the mitoxantrone dose corresponds to a roughly fourfold doxorubicin-equivalent dose. For instance, a patient receiving six cycles of MINE would receive an estimated total dose of 8 mg/m^2^ × 6 × 4 = 192 mg/m^2^ doxorubicin-equivalent. Adding the standard six cycles of prior R-CHOP (typically 50 mg/m^2^ per cycle), the cumulative exposure would be 192 mg/m^2^ + (6 × 50 mg/m^2^) = 492 mg/m^2^ doxorubicin-equivalent, which is indeed quite high [28]. Nevertheless, most patients did not require additional hospitalizations for supportive care, suggesting a favourable safety profile while avoiding the high toxicity also typically associated with platinum-based regimens.

The main limitations of this study include its retrospective design, a consequently notable proportion of missing data for some variables, and a relatively short median follow-up, despite the 17-year data collection period. These limitations are partly attributable to the advanced age and frailty of this patient population, as well as the aggressive character of this disease. Moreover, almost half of the cohort did not receive rituximab, largely due to its absence from departmental protocols at the time. This may have led to an underestimation of the regimen’s full efficacy, as the benefit of its inclusion is clearly established in the literature and was further corroborated by our findings [2]. On the other hand, the fact that approximately half of the patients did not receive rituximab was because they had received a rituximab-based regimen within the previous six months, which automatically renders a poor prognosis in this group. It is therefore difficult to determine whether patients treated solely with MINE had worse outcomes due to the absence of rituximab or because they were already refractory to the previous treatment line. Indeed, the potential prognostic value of rituximab was not retained in the multivariate analysis. Lastly, as previously mentioned, our cohort ultimately included patients who may be considered fit but were excluded from ASCT solely due to their age, in accordance with the institutional protocol.

## Conclusions

To our knowledge, this is the largest study to date focusing exclusively on the outcomes of the MINE regimen. While randomized prospective trials remain warranted, our findings reinforce MINE as a viable, cost-effective alternative for patients with R/R DLBCL who are ineligible for ASCT. Notably, the observed outcomes in our cohort are quite comparable to those reported with other commonly used salvage regimens, like R-GemOx and R-Pola-Benda. Furthermore, MINE avoids the additional toxicity associated with platinum agents and is generally well tolerated, with limited myeloablative effects, perhaps making it also a suitable option for an outpatient setting. Regarding newer targeted therapies, despite their promising efficacy, they often comprise a substantial financial burden and are not devoid of toxicity, such as profound and prolonged B-cell aplasia. Thus, while the current focus on targeted therapies is transforming the treatment landscape of R/R DLBCL, traditional chemotherapy protocols like MINE continue to offer meaningful clinical benefit and should not be overlooked, particularly in resource-limited settings or as a tailored individualized approach.
